# Unmet needs in ankylosing spondylitis patients receiving tumour necrosis factor inhibitor therapy; results from a large multinational real-world study

**DOI:** 10.1186/s41927-020-0118-z

**Published:** 2020-03-02

**Authors:** A. Deodhar, V. Strand, P. G. Conaghan, E. Sullivan, S. Blackburn, H. Tian, K. Gandhi, S. M. Jugl, R. Alten

**Affiliations:** 1 Oregon Health and Science University, Portland, OR USA; 2 Biopharmaceutical Consultant, Portola Valley, CA USA; 3 Leeds Institute of Rheumatic and Musculoskeletal Medicine, University of Leeds & NIHR Leeds Biomedical Research Centre, Leeds, UK; 4 Adelphi Real World, Bollington, UK; 5 Novartis Pharmaceuticals Corporation, East Hanover, NJ USA; 6 Novartis Pharma AG, Basel, Switzerland; 7 Schlosspark-Klinik, University Medicine, Berlin, Germany

**Keywords:** Ankylosing spondylitis, DMARD, Quality of life, Tumour necrosis factor-alpha, Treatment failure

## Abstract

**Background:**

Symptoms and comorbidities of ankylosing spondylitis (AS) considerably reduce health-related quality of life (HRQoL) and ability to work. This real-world study assessed rates of tumour necrosis factor inhibitor (TNFi) use and switching, treatment failure, and associations between failing TNFi and HRQoL, work productivity and activity impairment (WPAI).

**Methods:**

AS patients and their treating physicians completed questionnaires capturing patient demographics, clinical status, TNFi treatment history, reasons for switching TNFi, HRQoL and WPAI. Current TNFi was determined as “failing” if, after ≥3 months, physician-rated disease severity had worsened, remained severe, was “unstable/deteriorating”, physicians were dissatisfied with disease control and/or did not consider treatment a “success”.

**Results:**

The analysis included 2866 AS patients from 18 countries. Of 2795 patients with complete treatment data, 916 (32.8%) patients had never received TNFi therapy, 1623 (58.1%) patients were receiving their 1st TNFi and 200 (7.2%) patients had ever received ≥2 TNFi (treatment switch). Primary or secondary lack of efficacy were the commonest reasons for switching, and the mean delay in switching after primary lack of efficacy was 11.1 months. 232 (15.4%) patients on TNFi were currently “failing” who, compared to those with treatment success, reported poorer HRQoL: 5-dimension EuroQoL (EQ-5D-3 L): 0.63 vs. 0.78; Medical Outcomes Study Short-Form Health Survey version 2 (SF-36v2) mental component summary (MCS): 41.8 vs. 46.3; physical component summary (PCS): 40.2 vs. 45.1; impaired work productivity: 46.4% vs. 25.0%; and activity: 44.5% vs. 29.6%; all *P* < 0.001.

**Conclusions:**

Among AS patients, switching TNFi is uncommon and delayed by nearly 1 year despite primary lack of efficacy. Patients currently failing TNFi experience worse physical function, HRQoL and work productivity.

## Background

Ankylosing spondylitis (AS) is characterized by inflammatory back pain, fatigue and joint swelling from axial and peripheral articular manifestations as well as comorbidities such as uveitis, inflammatory bowel disease, psoriasis and osteoporotic fractures which pose a significant burden to the patient [1, 2]. In addition, spinal inflammation and structural damage can lead to stiffness or immobility from vertebral fusions [3]. These painful and often disabling clinical features have a detrimental effect on quality of life and mortality as well as burdening the patient and society with impaired ability to work, substantial healthcare costs and productivity loss [2, 4].

Early diagnosis is important to improve outcomes; however, diagnosis is often delayed from symptom onset by as much as 14 years in the USA and 6–8 years in Europe [5–7]. Diagnosis and treatment delays are correlated with increased radiological severity of AS [5, 8].

Primary pharmacologic treatment of AS are non-steroidal anti-inflammatory drugs, with biological Disease Modifying Anti-Rheumatic Drugs (bDMARDs) such as tumour necrosis factor inhibitors (TNFi) as a first-line bDMARD option, and the interleukin (IL)-17A inhibitors secukinumab and ixekizumab recommended for patients who have failed TNFi treatment [9]. It is not uncommon for patients with AS to experience TNFi therapeutic failure. For example, in a longitudinal, observational study of 249 cases of AS patients treated with TNFi, these agents had been discontinued at 1 year in 56 (22.5%) cases. Reasons for TNFi discontinuation included lack of efficacy and adverse events in 36.4 and 43.6% of cases, respectively [10]. Interestingly in this study, patients with RA had lower retention rates than patients with AS (65.4% vs 77.5%, respectively), which may reflect fewer options for alternative therapies in AS.

This multinational study was designed to describe use and switching of TNFi in AS patients; to assess rates of current TNFi failures and the associations of failing TNFi treatment with patient-reported health-related quality of life (HRQoL), work productivity and activity impairment (WPAI) using real-world data.

## Methods

The present study analysed data acquired from the Adelphi AS Disease Specific Program™ (DSP) from 2015 to 2016 in the following 18 countries: the USA, Canada (North America), Brazil, Mexico (LatAm), France, Germany, Italy, Spain, the UK (EU5), Japan, Malaysia, South Korea, Taiwan, Australia (APAC), Turkey, Egypt, Saudi Arabia and the United Arab Emirates (T&ME). DSPs are large multinational point-in-time surveys designed to identify current disease management, and both patient- and physician-reported disease impact from real-world clinical practice settings [11]. The DSP was conducted in line with contemporary legislation, namely the US Health Insurance Portability and Accountability Act 1996 [12] and the Health Information Technology for Economic and Clinical Health Act legislation [13]. DSPs also comply with relevant market research guidelines and legal obligations. Data collection was performed in accordance with the European Pharmaceutical Marketing Research Association guidelines and ethics committee approval was therefore not required [14]; i.e. DSPs are non-interventional and no personally identifiable protected health information is extracted, though all patients who participated provided informed consent.

### Patients and physicians

Eligible patients were adults over 18 years of age, had a physician-confirmed diagnosis of AS, were not currently involved in a clinical trial and visited a participating physician. Eligible physicians (rheumatologists, and orthopedists and internists in Japan) were those who were treating AS patients and practicing ≥3 years.

Participating physicians completed a questionnaire for 1 to 8 consecutive AS patients. Physicians reported patient demographics, clinical assessments, medication use and treatment history; responses were based on data available from treatment records. Patients were then invited to complete a voluntary patient-reported questionnaire. The questionnaires included the 5-dimension EuroQoL (EQ-5D-3 L) [15, 16], Medical Outcomes Study Short-Form Health Survey version 2 (SF-36v2) [17, 18] and WPAI General Health [19]. Questionnaires were completed independently from physicians and returned in sealed envelopes to ensure confidentiality.

### Definitions

**Fig. 1:**
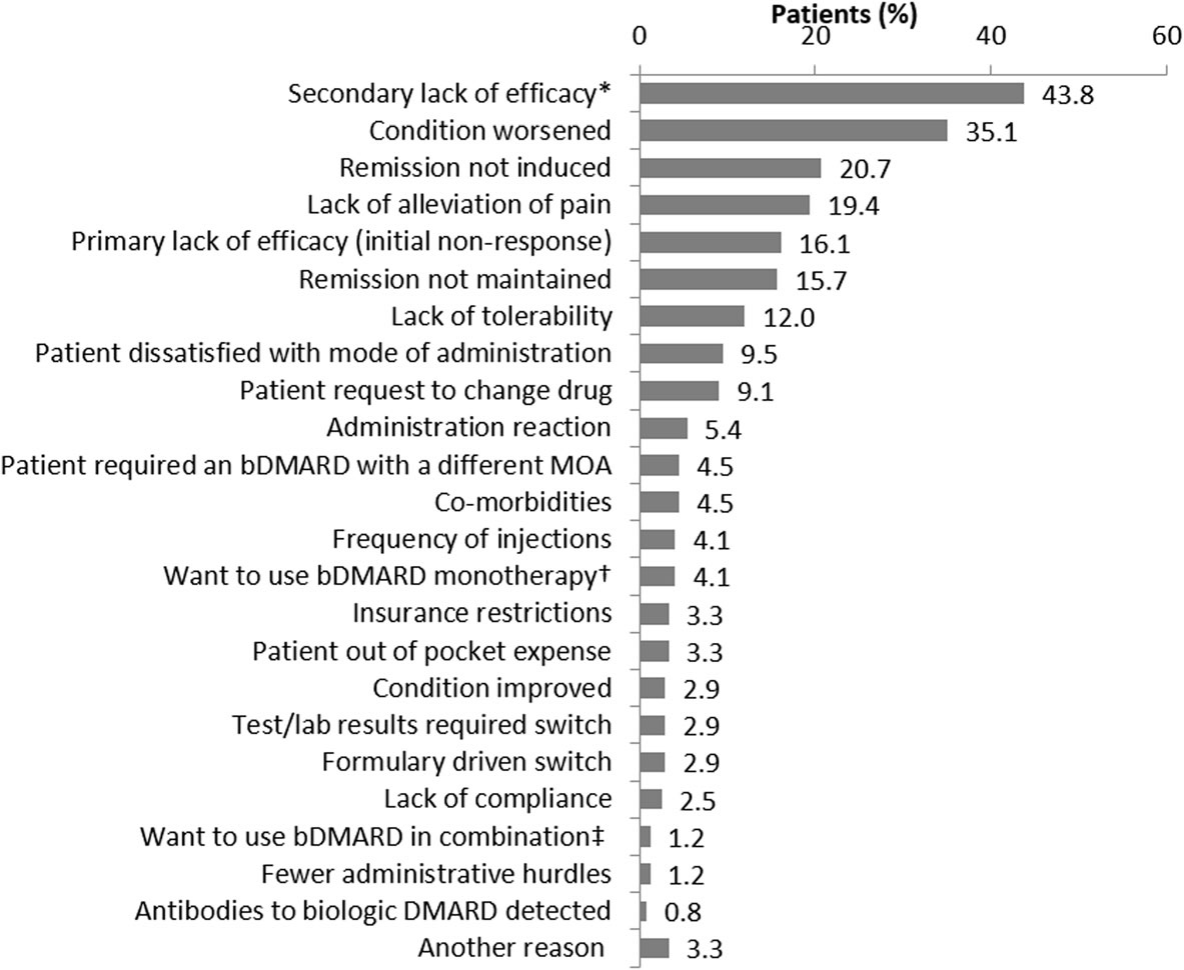
Physician-reported reasons given for patient switching from 1st to 2nd line TNFi. * Secondary lack of efficacy: loss of response over time; † I wanted to use bDMARD that can be used as a monotherapy; ‡ I wanted to use a bDMARD that can be used in combination. MOA: Mode of action; bDMARD: biologic Disease Modifying Anti Rheumatic Drug

To be eligible for assessment of treatment response patients had to be exposed to a TNFi ≥3 months. “TNFi switching” was defined as a change from one TNFi to another. The reasons for switching from 1st to 2nd TNFi were selected by physicians from a list of choices including primary lack of efficacy (initial non-response) or secondary lack of efficacy (loss of response over time), patient change (improvement, worsening of condition), lack of tolerability, patient preference, administrative reasons (i.e. formulary requirements) and physician preference for an alternative therapy; Fig. 1 provides the full list of options; no additional explanation of the options were provided so results represent physicians’ real-world interpretation of reasons.

Patients were defined as “failing TNFi” if, after ≥3 months of treatment with their current TNFi therapy, at least one of the following criteria (assessed by the treating physician) was met: disease severity (mild, moderate or severe) had worsened or remained severe; disease activity (improving, stable, unstable or deteriorating) was unstable or deteriorating; or physician reported dissatisfaction with current control of AS. Any patient not defined as “failing TNFi” was defined as “TNFi success”.

### Statistical analyses

Analyses were performed at both global and regional levels. Categorical variables are presented as counts and proportion of respondents. Continuous numerical variables are presented as means and standard deviations (SD). Pearson’s χ^2^ test was used to evaluate differences in failure rates by lines of TNFi.

Patient characteristics were analyzed descriptively and included demographics (age, gender and Body Mass Index [BMI]), human leukocyte antigen B27 (HLA-B27) status, number of TNFi therapies ever received, duration patients remained on 1st TNFi before switching, reasons for switching from 1st to 2nd TNFi, and proportions of patients currently failing TNFi overall and at each line of therapy.

Linear regression analyses were used for EQ-5D-3 L, SF-36v2 (physical component summary [PCS] and mental component summary [MCS]) scores and WPAI. Treatment response (failing TNFi or TNFi success) was the independent variable; differences in age, gender, BMI, smoking status, time since symptom onset and region were controlled for. Predicted values for all outcomes were subsequently stratified by TNFi failure or success. All other variables were fixed at their means.

Stata Statistical Software: Release 15 (StataCorp LP, College Station, TX) was used for all analyses.

## Results

### Patients characteristics

Six-hundred and forty physicians (North America, *n* = 97; LatAm, *n* = 31; EU5, *n* = 299; APAC, *n* = 115; T&ME, *n* = 98) and 2866 AS patients (North America, *n* = 538; LatAm, *n* = 139; EU5, *n* = 1512; APAC, *n* = 353; T&ME, *n* = 324) from 18 countries participated in the study; 1406 patients (49%) completed the voluntary patient questionnaires including EQ-5D-3 L (*n* = 1382), SF-36v2 (*n* = 1402) and WPAI (*n* = 1352).

Patient characteristics such as mean age, BMI, time since diagnosis, Bath Ankylosing Spondylitis Disease Activity Index (BASDAI) and Bath Ankylosing Spondylitis Functional Index were similar across most regions, although differences were noted mostly in T&ME patients (see Additional file 1: Table S1). In T&ME patients, mean age was < 40 years (37.5 years), whereas in all other regions the mean age was > 40 years. Mean time since symptom onset ranged from 2.8 years in T&ME to 10.8 years in North America, and mean time since diagnosis ranged from 1.5 years in T&ME to 7.3 years in North America. The proportion of patients classified as “severe” by their treating physician ranged from 3.6% in Latin America to 7.7% in T&ME. Median BASDAI scores ranged from 2.5 in T&ME to 4.0 in North America for the subset of patients in whom data was available.

### TNFi use and switching

Of the 2795 (of 2866) patients with complete treatment data, 916 (32.8%) had never received a TNFi, 1623 (58.1%) had received one, 200 (7.2%) two, and 56 (2.0%) three or more TNFi (Table 1). The mean (SD) disease durations for these groups of patients were 4.4 (7.2), 6.3 (7.3), 9.5 (7.7) and 13.0 (8.0) years, respectively.

**Table 1:** Patient TNFi therapy exposure and bDMARD switching

	All (*n* = 2795)	North America (*n* = 530)	LatAm (*n* = 137)	EU5 (*n* = 1478)	APAC (*n* = 335)	T&ME (*n* = 315)
Number of previous TNFi therapies ever received, n (%)						
0	916 (32.8)	108 (20.4)	15 (10.9)	535 (36.2)	103 (30.7)	155 (49.2)
1	1623 (58.1)	347 (65.5)	113 (82.5)	821 (55.5)	194 (57.9)	148 (47.0)
2	200 (7.2)	60 (11.3)	9 (6.6)	89 (6.0)	31 (9.3)	11 (3.5)
3+	56 (2.0)	15 (2.8)	0 (0.0)	33 (2.2)	7 (2.1)	1 (0.3)
Time on 1st bDMARD despite primary lack of efficacy, months	(*n* = 34)	(*n* = 16)		(*n* = 13)	(*n* = 2)	(*n* = 3)
Mean (SD)	11.1 (10.2)	12.6 (11.6)	–	10.7 (9.7)	1 (0.0)	11.3 (5.0)
Median	9.5	11.0	–	9.0	1.0	12.0
Min, Max	0.0–36.0	2.0–36.0	–	0.0–36.0	1.0–1.0	6.0–16.0
IQR	0.0, 1.0	0.0, 1.5	0.0, 0.0	0.0, 1.0	0.0, 0.5	0.0, 0.0

*Abbreviations: APAC* Asia Pacific region, *bDMARD* biological disease modifying anti-rheumatic drug; *BMI* body mass index, *EU5* European Union 5, *IQR* interquartile range, *LatAm* Latin America, *T&ME* Turkey and Middle East, *SD* standard deviation, *TNFi* tumour necrosis factor inhibitor

In 242 patients where information for switching from 1st to 2nd TNFi therapy was available, the commonest reason was lack of efficacy in over half of patients. Secondary lack of efficacy (loss of response over time) was reported in 106 (43.8%) patients, and primary lack of efficacy (initial non-response) in 39 patients (16.1%) (Fig. 1). Other reasons for switching from 1st TNFi therapy were “condition worsened” (*n* = 85; 35.1%), “remission not induced” (*n* = 50; 20.7%), “lack of alleviation of pain” (*n* = 47; 19.4%) and “remission not maintained” (*n* = 38; 15.7%).

Information on treatment duration was available for 34 of the 39 patients who switched therapy due to primary lack of efficacy. These patients remained on the failed therapy for a mean (SD) of 11.1 months (10.2) before treatment was switched. The longest reported time to switch was 36 months (Table 1).

### Patients currently “failing TNFi”

**Fig. 2:**
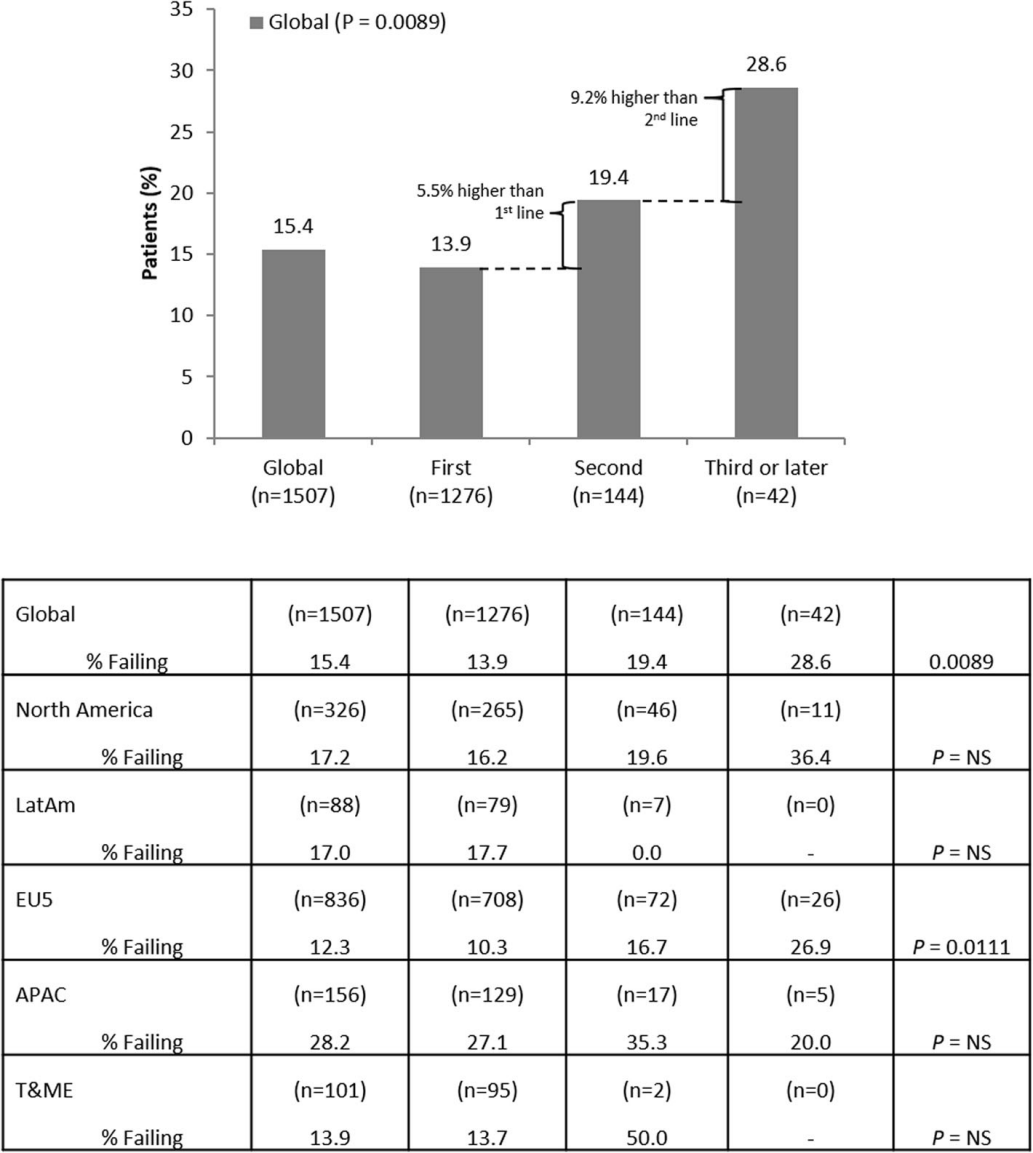
Rates of TNFi failure on successive lines of TNFi therapy. APAC, Asia Pacific region; EU5, European Union 5; T&ME, Turkey and Middle East

Treatment success or failure was evaluated in 1507/2866 patients. A total of 916 patients were excluded as they had never received TNFi, 90 were not currently receiving TNFi, 182 were exposed to TNFi < 3 months, 100 had missing duration of TNFi treatment data and 71 had other missing treatment data. Globally, 232 (15.4%) patients were failing current TNFi according to the definitions provided and failure rates were higher with each successive TNFi. Among patients with data available on the number of TNFi ever received, 13.9% were failing their 1st TNFi. Globally, failure rates increased to 28.6% in patients receiving their 3rd (or later) TNFi (*P* = 0.0089). In North America and EU5, 16.2% (North America) and 10.3% (EU5) of patients were failing 1st TNFi, and 36.4% (North America; *P* = 0.2062) and 26.9% (EU5; *P* = 0.0111) were failing their 3rd (or later) TNFi. For regions with lower patient numbers, the trend again increased with subsequent TNFi (either 2nd or 3rd, if available) (Fig. 2).

### Patient characteristics by failing TNFi and TNFi success groups

With some exceptions, patient characteristics between failing and successful TNFi groups were similar in most regions (Table 2). Patients with treatment success were more likely male in EU5 but no other regions (Table 2). Globally, patients who were failing TNFi had more AS symptoms compared with TNFi successes (2.5 vs. 1.8, *P* < 0.0001), as observed in North America (3.1 vs. 2.1; *P* = 0.0003), EU5 (2.4 vs. 1.6; *P* < 0.0001) and APAC (2.3 vs. 1.8, *P* = 0.0203) but not in LatAm or T&ME (Table 2). Globally, both erythrocyte sedimentation rate (ESR) (24.9 vs. 15.1; *P* < 0.0001) and C-reactive protein (CRP) (7.2 vs. 4.5; *P* < 0.0001) levels were higher in patients failing compared with TNFi treatment success groups (Table 2).

**Table 2:** Patient characteristics of “TNFi success” and “failing TNFi” cohorts

	All		North America		LatAm		EU5		APAC		T&ME	
Group	TNFi Success (*n* = 1275)	Failing TNFi (*n* = 232)	TNFi Success (*n* = 270)	Failing TNFi (*n* = 56)	TNFi Success (*n* = 73)	Failing TNFi(*n* = 15)	TNFi Success (*n* = 733)	Failing TNFi (*n* = 103)	TNFi Success (*n* = 112)	Failing TNFi (*n* = 44)	TNFi Success (*n* = 87)	Failing TNFi (*n* = 14)
Age, years												
Median	43.0	45.0	45.0	47.0	41.0	45.0	44.0	49.0	40.0	36.5	38.0	34.0
Mean (SD)	43.8 (12.1)	45.7 (13.2)	45.5 (13.4)	47.8 (12.0)	42.5 (12.0)	44.8 (6.3)	44.6 (11.8)	48.8 (13.8)	40.6 (12.4)	39.3 (13.5)	37.8 (6.9)	35.9 (4.7)
*P*	0.0516		0.1996		0.0956		0.0035		0.5310		0.2808	
Male, n (%)	1009 (79.1)	165 (71.1)	220 (81.5)	42 (75.0)	50 (68.5)	9 (60.0)	582 (79.4)	66 (64.1)	90 (80.4)	36 (81.8)	67 (77.0)	12 (85.7)
*P*	0.0097		0.2708		0.5554		0.0009		1		0.7287	
BMI, kg/m^2^												
Median	25.3	25.2	25.9	27.0	25.6	25.0	25.2	25.6	23.4	22.9	25.6	25.7
Mean (SD)	25.7 (3.8)	26.0 (4.7)	26.5 (4.1)	27.8 (4.9)	25.9 (3.2)	25.2 (5.0)	25.5 (3.4)	26.2 (4.5)	23.8 (4.3)	23.4 (4.0)	26.8 (4.7)	26.5 (4.0)
*P*	0.8563		0.0418		0.3570		0.2809		0.4638		0.9881	
Current Smoker												
n, %	381 (29.9)	81 (34.9)	37 (13.7)	17 (30.4)	7 (9.6)	2 (13.3)	252 (34.4)	38 (36.9)	29 (25.9)	17 (38.6)	56 (64.4)	7 (50.0)
*P*	0.1412		0.0049		0.6471		0.6586		0.1235		0.3761	
Time since symptom onset (years)												
n	1110	196	238	43	73	15	611	86	103	38	85	14
Median	7.0	8.0	8.0	10.0	5.0	3.0	9.0	10.0	8.0	5.0	2.3	2.0
Mean (SD)	10.4 (9.2)	11.7 (11.2)	12.0 (10.8)	11.6 (9.1)	8.9 (9.7)	8.3 (10.9)	10.8 (8.8)	14.5 (12.6)	10.8 (8.4)	9.9 (10.4)	3.0 (2.1)	3.3 (2.7)
*P*	0.6081		0.7041		0.2977		0.0554		0.1978		0.8485	
Time since diagnosis (years)												
n	1170	209	253	51	72	15	659	91	103	38	83	14
Median	5.0	5.0	5.0	5.0	3.0	2.0	5.0	6.0	5.0	3.0	1.2	1.0
Mean (SD)	7.2 (7.2)	8.4 (9.6)	7.9 (8.1)	9.0 (9.3)	5.9 (6.4)	6.6 (10.5)	7.8 (7.0)	10 (10.3)	7.2 (6.7)	6.7 (8.5)	1.7 (1.8)	2.1 (2.2)
*P*	0.7489		0.2647		0.3055		0.1946		0.0886		0.7456	
HLA-B27 + ve												
n	919	145	204	35	63	12	542	69	65	22	45	7
n, %	809 (88.0)	129 (89.0)	177 (86.8)	32 (91.4)	57 (90.5)	12 (100.0)	484 (89.3)	59 (85.5)	55 (84.6)	20 (90.9)	36 (80.0)	6 (85.7)
*P*	0.8900		0.5861		0.5811		0.3159		0.7224		1	
Current AS symptoms												
n	1275	232	270	56	73	15	733	103	112	44	87	14
Median	1.0	2.0	2.0	2.5	2.0	1.0	1.0	2.0	1.0	2.0	1.0	2.0
Mean (SD)	1.8 (1.4)	2.5 (1.7)	2.1 (1.7)	3.1 (2.2)	1.9 (1.4)	1.7 (1.2)	1.6 (1.3)	2.4 (1.6)	1.8 (1.6)	2.3 (1.4)	1.5 (1.5)	1.9 (1.4)
*P*	< 0.0001		0.0003		0.6424		< 0.0001		0.0203		0.2567	
ESR, mm/hr (within 3 months)												
n	780	143	148	36	43	7	438	57	79	29	72	14
Median	12.0	20.0	14.0	31.5	10.0	12.0	12.0	21.0	10.0	15.0	20.0	32.0
Mean (SD)	15.1 (11.8)	24.9 (19.3)	17.8 (12.8)	27.4 (19.0)	11.5 (7.8)	11.1 (8.5)	13.8 (11.6)	22.5 (16.3)	13.0 (12.0)	28.4 (26.8)	22.0 (8.8)	28.3 (13.7)
*P*	< 0.0001		0.0078		0.9551		< 0.0001		0.0035		0.1976	
CRP, mg/l (within 3 months)												
n	718	124	116	24	35	4	425	54	74	28	68	14
Median	2.5	5.0	1.6	3.3	2.0	1.0	3.0	6.0	1.1	3.0	2.6	4.9
Mean (SD)	4.5 (6.0)	7.2 (9.0)	4.6 (8.2)	7.7 (9.5)	3.2 (4.0)	0.8 (0.5)	4.7 (5.1)	9.8 (11.2)	3.7 (7.5)	3.6 (3.3)	4.2 (5.9)	5.0 (0.9)
*P*	< 0.0001		0.0167		0.1117		< 0.0001		0.3160		0.0042	

Patients were deemed to be failing TNFi after at least 3 months if disease severity had worsened or remained severe, disease activity was unstable or deteroriating, disease was not considered by physician to be controlled, nor treatment a success. Patients not considered to be failing TNFi were considered to be “TNFi success”

*Abbreviations: APAC* Asia Pacific region, *AS* ankylosing spondylitis, *BMI* body mass index, *CRP* c-reactive protein, *ESR* erthyrocyte sedimentation rate, *EU5* European Union 5, *HLA-B27* human leukocyte antigen B27, *LatAm* Latin America, *T&ME* Turkey and Middle East, *SD* standard deviation

### Association between failing current TNFi and HRQoL and WPAI

**Fig. 3:**
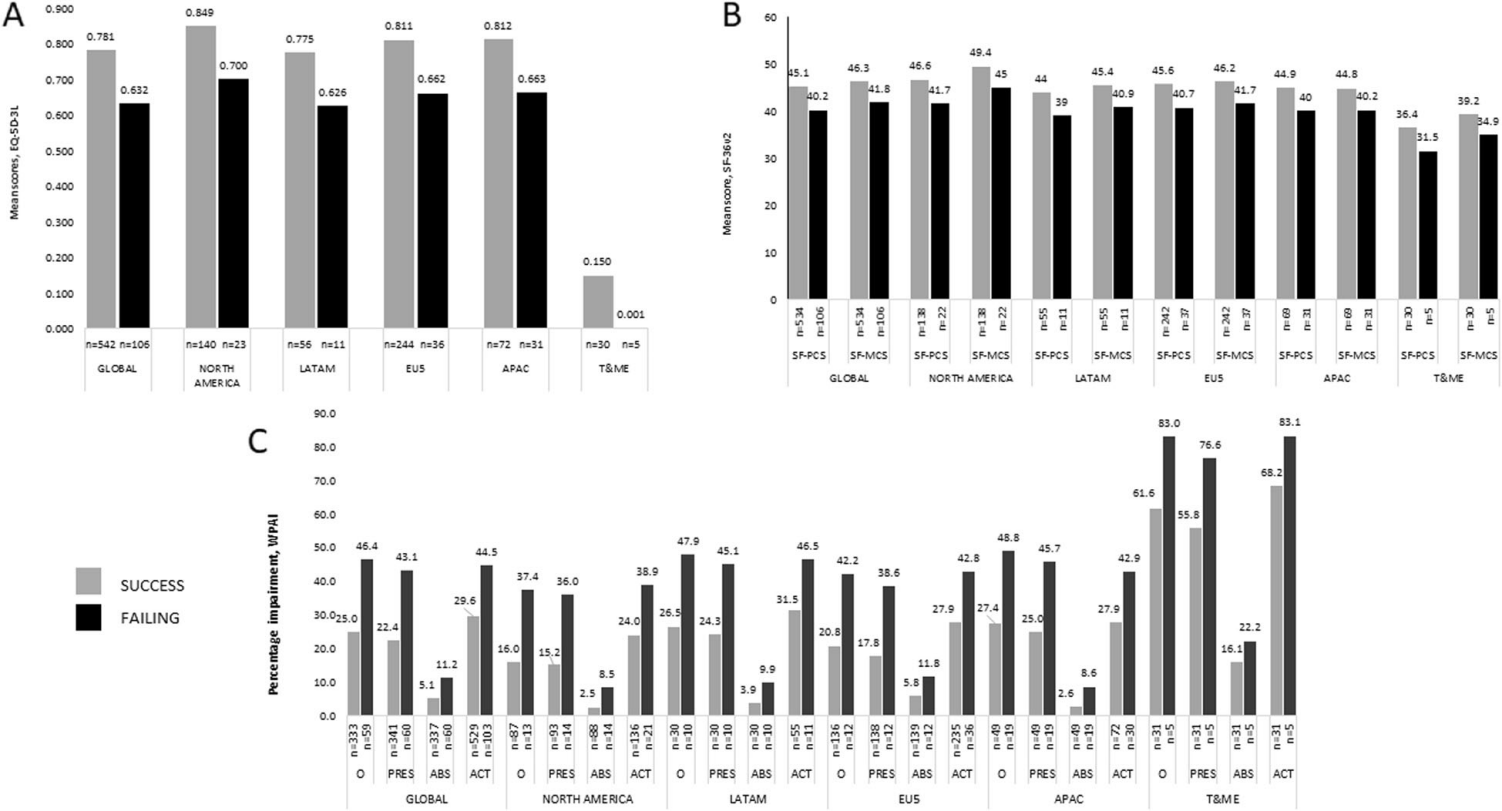
Results are adjusted for age, gender, smoking status, BMI, time since onset of symptoms and region. ABS, absenteeism; ACT, activity impairment; APAC, Asia Pacific region; EU5, European Union 5; LatAm, Latin America; O, overall work impairment; PRES, presenteeism; SD, standard deviation; T&ME, Turkey and Middle East. SF-PCS, *P* < 0.0001; SF-MCS, *P* = 0.0004; overall work impairment, *P* < 0.0001; presenteeism, *P* < 0.0001; absenteeism, *P* = 0.0073; activity impairment, *P* < 0.0001

**Fig. 4:**
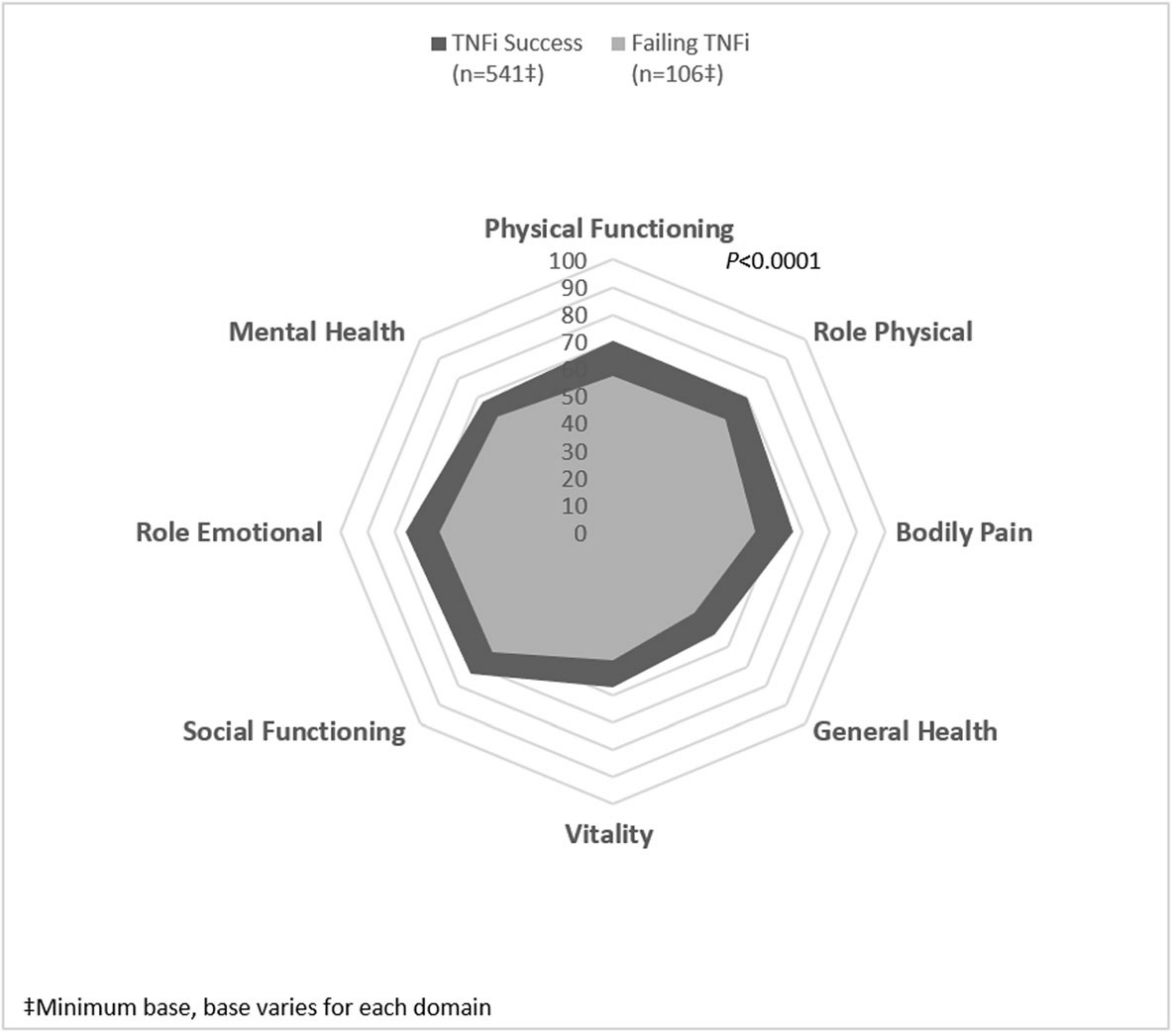
Results are adjusted for age, gender, BMI, smoking status, time since symptom onset and region

Linear regression analysis exposed that failing treatment compared with treatment success was associated with a lower HRQoL, shown by the impact on the adjusted EQ-5D-3 L (0.63 vs. 0.78, coef. -0.149, *P* < 0.0001), and SF-36v2 PCS (40.2 vs. 45.1, coef. -4.917, *P* < 0.0001) and MCS scores (41.8 vs. 46.3, coef. -4.511, *P* < 0.0001) (Fig. 3a and b). All SF-36 domain scores were lower among patients failing TNFi treatment compared with those with treatment success (Fig. 4). Among those working, WPAI overall work productivity was confirmed as worse in patients failing vs. not failing (46.4% vs. 25.0%, coef. 21.397, *P* < 0.0001), as was absenteeism (11.2% vs. 5.1%, coef. 6.035, *P* = 0.007) and presenteeism (43.1% vs. 22.4%, coef. 20.758, *P* < 0.0001), and impairment in daily activities in the entire population (44.5% vs. 29.6%, coef. 14.961, *P* < 0.0001) (Fig. 3c).

## Discussion

This real-world, large multinational study of TNFi use in patients with AS demonstrates that TNFi do not consistently deliver sustained efficacy; switching was mainly associated with primary and secondary treatment failures, i.e. primary and secondary lack of efficacy, and many patients were failing their current TNFi. Clinical responses to TNFi declined with each subsequent treatment, evidenced by a higher incidence currently failing their 2nd or 3rd TNFi. Our cross-sectional data analysis allows us to report the rates of patients currently failing therapy they are still taking, based on their clinical profile. This differs from previous studies [20–22], where failure rates were calculated based on the proportion of patients who switched therapy as an indicator of failure.

The most common reasons for switching in our study were secondary and primary lack of efficacy (43.8 and 16.1%, respectively), worsening of condition (35.1%), remission not induced or maintained (20.7 and 15.7%, respectively), and lack of alleviation of pain (19.4%) and lack of tolerability (12.0%), consistent with previous reports [23, 24]. Data reported in our study reflect physicians’ responses, and thus their real-world reasoning for switching therapy, even though there may be potential overlaps in the responses. A recent review from 21 studies reported the most common reasons for switching to a 2nd TNFi were lack of efficacy (14–68%), loss of efficacy (13–61%) and adverse events/poor tolerability (13–57%) [25]. Lack of efficacy or adverse events (AE) after the 1st TNFi may justify switching to another TNFi on the basis that they differ in structure, immunogenicity, half-life and administration schedules [23, 24]. This is supported by the common observation that patients who switched because of loss of response over time, due to adverse events or other reasons, were more likely to respond to a 2nd TNFi than patients with a primary (initial) lack of response [25].

In cases where physicians reported switching from 1st to 2nd TNFi therapy was due to primary lack of efficacy, time to switch to the 2nd TNFi occurred at 11.1 months on average. Our global findings are consistent with data from a retrospective analysis of patients treated with TNFi at two British hospitals where the mean duration of treatment prior to switching in patients with inadequate efficacy was 11 months [26]. These findings indicate that patients may be maintained on failing therapy for significantly longer than the 12 weeks recommended by ACR/SAA/SPARTAN treatment guidelines [9].

Our analyses also demonstrate that failing TNFi therapy is associated with poorer patient-reported HRQoL, as measured by EQ-5D-3 L and SF-36, as well as negative impact on daily activities measured by WPAI. Analysis of SF-36 highlighted that failing TNFi treatment is associated with poorer outcomes across all domains associated with HRQoL, despite the fact that differences between patient groups in MCS and PCS scores were not large. Future studies are needed to confirm our results and examine the clinical relevance of these findings. Although absenteeism and presenteeism are common in patients with AS, which has been shown to negatively impact HRQoL [4, 27], to the best of our knowledge, this is the first real-world study to compare HRQoL and WPAI in patients with successful vs. failing TNFi treatment. The impact of time to switch to another TNFi on HRQoL, and economic and social burden of AS, was not explored in this study but it is an area of interest for future research.

TNFi treatment failure is an important consideration in the management of AS patients, since until recently TNFi were the only approved biologic treatment for AS. Following the recent approvals of the IL-17A antagonists secukinumab and ixekizumab, the ACR/SAA/SPARTAN recommendations include switching to either [9] as they are demonstrated effective in patients with inadequate response to TNFi, providing an option for those whose disease is not controlled by TNFi therapy [28, 29]. At the time of this study, TNFi were the only biologic therapy available, therefore the real-world clinical impact of switching to a new class of biologics such as secukinumab or ixekizumab could not be assessed and represents an important area of future research.

A major strength of this study is that it presents real-world data in patients with a clinically confirmed diagnosis of AS around the world, providing insight into rates of TNFi use and switching, as well as the negative impact on patients’ HRQoL and work productivity associated with failing TNFi therapy.

Several potential limitations associated with data derived from this cross-sectional, real-world study should be considered. A primary limitation of the analysis is that the source data is a point-in time survey and does not capture the exact timepoint at which patients fail to respond to therapy, therefore it was necessary to rely on physician-reported reasons for switching therapy to identify the subset who failed to respond. Cross-sectional studies are limited in their selection of patients, sample size and data collection. The high rate of patients receiving TNFi in countries where access to biologics is limited (LatAm) may be impacted by small physician and patient sample size and reflect a selection bias. In contrast to a clinical trial where disease severity and activity are assessed by validated measures, physician ratings of disease severity/activity may be considered subjective, and hence a limitation. However, our study reflects how physicians’ practice in a real-world clinical setting where assessments may be more holistic rather than focusing solely on disease activity. Finally, although recall bias is a common limitation of surveys, as data were collected at the time of patients’ appointments, the likelihood of recall bias is reduced.

## Conclusions

In conclusion, this large multinational real-world study of AS patients demonstrated that lack or loss of efficacy of TNFi is common, yet it appears that patients failing the 1st TNFi (primary lack of efficacy/failure) are not switched to another TNFi for nearly 1 year. A significant proportion of patients who failed their 1st TNFi did not respond to subsequent TNFi. Failing TNFi therapy is associated with poorer HRQoL, physical activity and work productivity. Whether regular monitoring and earlier use of appropriate therapies upon identification of lack of efficacy leads to improvements in symptom control and HRQoL remains to be seen.

## Supplementary information

**Additional file 1: Table S1.** Patient characteristics, Description of data: Detailed patient characteristics, including disease severity and disease scores, are provided.

## Data Availability

Data are owned by Adelphi Real World. All requests regarding data should be addressed directly to Adelphi Real World.

## Abbreviations

APAC: Asia-Pacific region
AS: Ankylosing spondylitis
BASDAI: Bath ankylosing spondylitis disease activity index
bDMARD: Biological disease modifying anti-rheumatic drug
BMI: Body mass index
CRP: C-reactive protein
DSP: Disease Specific Program
EQ-5D-3 L: 5-dimension EuroQoL
ESR: Erythrocyte sedimentation rate
EU5: European Union 5
HLA-B27: Human leukocyte antigen B27
HRQoL: Health-related quality of life
IL-17A: interleukin-17A
IQR: Interquartile range
LatAm: Latin America
MCS: Mental component summary
PCS: Physical component summary
SD: Standard deviation
SF-36v2: Short-form health survey version 2
T&ME: Turkey and Middle East
TNFi: Tumor necrois factor inhibitor
USA: United States of America
WPAI: Work productivity and activity impairment
