# Supplementary material for: Unmet needs in ankylosing spondylitis patients receiving tumour necrosis factor inhibitor therapy; results from a large multinational real-world study
Source: BMC Rheumatol. 2020 Mar 2;4:19. doi: 10.1186/s41927-020-0118-z (PMC7050131; doi:10.1186/s41927-020-0118-z)
Supplement: Supplementary file 1 — Additional file 1: Table S1. Patient characteristics, Description of data: Detailed patient characteristics, including disease severity and disease scores, are provided. [file 41927_2020_118_MOESM1_ESM.docx]

**Supplementary Table 1.** Patient characteristics

| **Characteristic** | **All (n=2866)** | **North America (n=538)** | **LatAm (n=139)** | **EU5**  **(1512)** | **APAC (n=353)** | **T & ME  (n=324)** |  |
| --- | --- | --- | --- | --- | --- | --- | --- |
| **Age, years** | **(n=2863)** | **(n=538)** | **(n=139)** | **(n=1512)** | **(n=350)** | **(n=324)** |  |
| Median  IQR  Mean (SD) | 42.0  35.0, 52.0  43.7 (12.7) | 45.0  35.0, 56.0  45.9 (14.0) | 43.0  37.0, 50.0  44.2 (10.9) | 44.0  35.0, 54.0  44.8 (12.6) | 40.0  32.0, 50.0  41.7 (13.6) | 37.0  34.0, 40.0  37.5 (6.8) |  |
| **Male, n (%)** | **2124 (74.1)** | **416 (77.3)** | **88 (63.3)** | **1094 (72.4)** | **283 (80.2)** | **243 (75.0)** |  |
| **Mean BMI, (kg/m^2^)** | **(n=2859)** | **(n=538)** | **(n=139)** | **(n=1512)** | **(n=348)** | **(n=322)** |  |
| Median  IQR  Mean (SD) | 25.2  23.1, 27.6  25.7 (4.0) | 26.3  24.2, 28.5  26.9 (4.3) | 25.4  23.4, 27.8  25.6 (3.5) | 25..0  23.1, 27.2  25.4 (3.5) | 23.3  21.1, 25.2  23.6 (4.1) | 25.9  24.4, 28.4  27.2 (4.9) |  |
| **Time since symptom onset (years)** | **(n=2460)** | **(n=461)** | **(n=138)** | **(n=1247)** | **(n=307)** | **(n=307)** |  |
| Median | 5.0 | 8.0 | 4.0 | 7.0 | 7.0 | 2.0 |  |
| IQR | 2.0, 12.0 | 4.0, 15.0 | 2.0, 13.0 | 3.0, 14.0 | 3.0, 15.0 | 1.0, 3.0 |  |
| Mean (SD) | 9.2 (9.6) | 10.8 (10.3) | 8.2 (9.5) | 10.0 (9.8) | 10.1 (9.5) | 2.8 (2.9) |  |
| **Time since diagnosis (years)** | **(n=2594)** | **(n=491)** | **(n=136)** | **(n=1342)** | **(n=315)** | **(n=310)** |  |
| Median | 3.0 | 4.0 | 2.0 | 4.0 | 4.0 | 0.8 |  |
| IQR | 1.0, 8.0 | 2.0, 10.0 | 1.0, 6.0 | 2.0, 10.0 | 1.0, 9.0 | 0.4, 1.9 |  |
| Mean (SD) | 6.2 (7.6) | 7.3 (8.6) | 5 (6.5) | 6.8 (7.7) | 6.6 (7.7) | 1.5 (2.3) |  |
| **Current severity (physician rated), n (%)** | **(n=2866)** | **(n=538)** | **(n=139)** | **(n=1512)** | **(n=353)** | **(n=324)** |  |
| Mild | 1734 (60.5) | 318 (59.1) | 80 (57.6) | 944 (62.4) | 239 (67.7) | 153 (47.2) |  |
| Moderate | 951 (33.2) | 186 (34.6) | 54 (38.8) | 474 (31.3) | 91 (25.8) | 146 (45.1) |  |
| Severe | 181 (6.3) | 34 (6.3) | 5 (3.6) | 94 (6.2) | 23 (6.5) | 25 (7.7) |  |
| **BASDAI at current consultation (physician reported)** | **(n=1001)** | **(n=28)** | **(n=45)** | **(n=653)** | **(n=171)** | **(n=104)** |  |
| Median | 3.0 | 4.0 | 3.0 | 2.8 | 3 | 2.5 |  |
| IQR | 1.8, 4.2 | 2.0, 5.8 | 2.0, 6.0 | 1.9, 4.0 | 1.6, 4.5 | 1.0, 5.0 |  |
| Mean (SD) | | 3.2 (2.1) | 3.9 (2.2) | 3.7 (2.5) | 3.1 (2.1) | 3.3 (2.3) | 3.1 (2.0) |
| **BASFI at current consultation (physician reported)** | **(n=332)** | **(n=31)** | **(n=13)** | **(n=240)** | **(n=32)** | **(n=16)** |  |
| Median | 3.0 | 4.1 | 3.0 | 3.0 | 3.6 | 4.0 |  |
| IQR | 2.0, 5.0 | 4.0, 5.0 | 2.0, 7.0 | 2.0, 4.0 | 1.9, 6.3 | 2.5, 5.0 |  |
| Mean (SD) | 3.6 (3.3) | 4.5 (1.2) | 4.2 (3.1) | 3.2 (2.1) | 5.2 (8.6) | 4.1 (1.7) |  |

* BASDAI and BASFI data is available for patients whose physician completed the assessment at the reference consultation. APAC, Asia Pacific region; BASDAI, Bath Ankylosing Spondylitis Disease Activity Index; BASFI, Bath Ankylosing Spondylitis Functional Index; BMI, body mass index; EU5, European Union 5; EQ-5D, EuroQol 5-dimensions questionnaire; LatAm, Latin America; T & ME, Turkey & Middle East; SD, standard deviation.
